# Nasal carriage rate and multiple antimicrobial resistance indices of *Staphylococcus aureus* among healthcare students at the Ahmadu Bello University, Nigeria

**DOI:** 10.4102/ajlm.v14i1.2667

**Published:** 2025-06-27

**Authors:** Sumayya Abdullahi, Idris N. Abdullahi, Hafeez A. Adekola, Nicholas Baamlong, Amos Dangana, Yahaya Usman, Abdurrahman E. Ahmad, Sumaiya Salisu, Mukhtar M. Abdulaziz

**Affiliations:** 1 Department of Medical Laboratory Science, Ahmadu Bello University, Zaria, Nigeria; 2 Department of Molecular Biology and Biotechnology, Nigeria Institute of Medical Research, Lagos, Nigeria; 3 Department of Family Medicine, University of Abuja Teaching Hospital, Abuja, Nigeria; 4 National Reference Laboratory, Nigeria Center for Disease Control and Prevention, Abuja, Nigeria; 5 Department of Medical Microbiology, Ahmadu Bello University, Zaria, Nigeria

**Keywords:** *Staphylococcus aureus*, multidrug resistance, multiple antibiotic resistance index, medical students, Nigeria

## Abstract

**Background:**

Healthcare students could harbour multidrug-resistant (MDR) and methicillin-resistant *Staphylococcus aureus* (MRSA). There is a need to understand the extent and factors associated with nasal carriage of these strains.

**Objective:**

This study determined the frequency and risk of nasal *S. aureus,* and multiple antimicrobial resistance indices among students at Ahmadu Bello University, Zaria, Nigeria.

**Methods:**

This comparative cross-sectional study collected nasal samples from 02 January 2024 to 31 July 2024 from healthcare students at Ahmadu Bello University, Nigeria, which were processed for *S. aureus* identification. Antimicrobial resistance phenotype was determined by the disk diffusion method. Structured questionnaires were used to collect participants’ sociodemographic and risk factor data.

**Results:**

A total of 251 students participated, including 126 (50.2%) men and 125 (49.8%) women (aged 17–44 years). The nasal carriage of *S. aureus* was 31.5% (79/251) and MRSA was 23.5% (59/251). Clinical-phase students had a higher frequency of nasal MRSA (25%) than preclinical-phase students (22.1%). *Staphylococcus aureus* resistance against non-beta-lactams was highest for tetracycline (49.4%) and ciprofloxacin (29.1%), with 39.2% (31/79) showing MDR. Medical and pharmacy students had statistically significant higher nasal carriage of MDR-*S. aureus* (*p* < 0.05). Students residing in households of 5–8 individuals had the highest nasal MDR-*S. aureus* carriage (*p* = 0.0044). *Staphylococcus aureus* isolates with multiple antimicrobial resistance indices of 0.2 (29.1%) and 0.3 (24%) were the most predominant.

**Conclusion:**

High levels of nasal MRSA and MDR-*S. aureus* were obtained from this study. The predominance of strains with high antimicrobial resistance indicates sources with high antibiotic use.

**What this study adds:**

To our knowledge, this is the first epidemiological study on the multiple antimicrobial resistance indices of nasal *S. aureus* in healthcare students in Africa. Moreover, this is the first report to categorises subgroup variation of nasal MDR-*S. aureus* carriage by the six major groups of healthcare students.

## Introduction

*Staphylococcus aureus* is a member of the nasal microbiota, and a leading source of healthcare- and community-associated infections in people, especially the methicillin-resistant *S. aureus* (MRSA) strains, resulting in morbidity and death.^1^ *Staphylococcus aureus* and MRSA may colonise a variety of places, including the skin and mucosal surfaces of humans and animals, and can contaminate environmental surfaces, food, and water.^1,2,3,4,5^ It is often present in healthy individuals without producing illness, but it is a risk factor for developing staphylococcal infections.^6^ In the healthcare setting, MRSA is of substantial concern, as it can be transmitted across patients, healthcare workers, students, and visitors.^7,8^

When MRSA is transmitted to immunocompromised patients, it can cause a variety of illnesses, ranging from minor skin infections to serious disorders such as endocarditis, osteomyelitis, and sepsis.^9^ Often, the overuse and misuse of antimicrobial agents selects for the emergence of MRSA and multidrug-resistant (MDR)-*S. aureus* (strains that are resistant to three or more classes of antimicrobial agents).^10^ These can complicate the use of antimicrobial chemotherapy against infectious diseases caused by *S. aureus* when the strains present with the MDR phenotype.^10^

Healthcare centres generally have sick individuals (humans or animals).^11^ Hence, students who receive clinical training in these settings could be at high risk of contracting clinically resistant *S. aureus* strains.^12^ Conversely, students colonised with MRSA in their nasal passages have the potential to transmit these strains to sick people and animals (for veterinary students) in healthcare centres during clinical posting and ward rounds,^8,13^ consequently increasing the burden or morbidity to the patients. Nevertheless, MRSA can also be acquired from both the community and animals (zoonosis).^1,2^

Healthcare students can harbour MDR and MRSA in their nostrils and can be the source of transmission to hospitalised patients and other individuals in healthcare facilities. It has been shown that nasal colonisation by *S. aureus* among these categories of students could be very high.^8^ Collectively, this indicates the need for stringent infection prevention and control measures among medical and health sciences students. It is important to note that these colonisation rates could vary, depending on the duration of contact with hospital settings, and on the type or form of contact with patients. Hence, the need to undertake the present study to determine the level of the indicated epidemiological parameters among various groups of healthcare students (either preclinical or clinical phases).

There are numerous studies, including many from most African countries and cities, that have identified the ecology of MRSA in medical students, as indicated in a systematic review and meta-analysis.^13^ In the global meta-analysis, a pooled prevalence of 28% nasal MRSA carriage in medical students was estimated.^13^ Furthermore, the study found out that a higher pooled prevalence was obtained in students at the clinical phase of training (33%) than in those at the preclinical phase of training (25%).^13^ However, there are very scarce data on subgroup analyses of MRSA carriage rates among students from the other major healthcare disciplines, and the multiple antimicrobial resistance (MAR) indices of the isolates.

This comparative cross-sectional study aims to determine the nasal carriage of *S. aureus,* its antimicrobial resistance phenotypes and MAR indices, and to assess the factors that could be associated with nasal *S. aureus* and MDR-*S. aureus* carriage among medical, veterinary, and health sciences students at Ahmadu Bello University, Zaria, Nigeria.

## Methods

### Ethical considerations

All study protocols were submitted for ethical review and were approved by the human research ethical committee of Ahmadu Bello University Teaching Hospital (approval no.: NHREC/TR/ABUTH-NHREC/01/02/23). All data generated were treated with the utmost confidence and analysed anonymously. All participants gave written informed consent before enrolment into the study. Samples and data collection from participants were performed according to Helsinki’s declaration as revised in 2024. Data collected were stored in a password-protected computer, only accessible by the principal investigator.

### Study area

This study was conducted from 02 January 2024 to 31 July 2024 at the Ahmadu Bello University and Ahmadu Bello University Teaching Hospital, Kaduna State, Nigeria. The university and teaching hospital serve as the main tertiary institution and reference hospital in the Northwest Geopolitical Zone of Nigeria. Moreover, they are the oldest and largest institutions for the training of medical and other healthcare professionals in Northern Nigeria.

### Study design, sample size, data collection

This was a cross-sectional study, and the sample size was calculated from the only available previous cross-sectional study on a healthy population of medical students in Nigeria.^14^ Using the 14% prevalence of nasal *S. aureus* from the study of Adesida et al.,^14^ a minimum sample size of 190 was calculated. However, for statistical credence, 251 healthy students who consented to participate were enrolled. Sociodemographic variables and other data such as prior use of antibiotics, household or hostel room density, and prolonged contact with animals were collated from the participants through structured, paper-based questionnaires administered by the researchers (interviewers).

### Sample collection

Nasal samples from both nostrils of eligible participants were collected by some of the investigators in batches and preserved in Amies transport media (HiMedia Laboratories, Maharashtra, India). Within 4 h of collection, the nasal samples were transported to the laboratory on cold packs (2 °C – 8 °C) by some of the investigators, where they were processed for *S. aureus* recovery. Isolation and identification of isolates were carried out based on the colonies’ colour, size, shape, tube coagulase test, strong mannitol fermentation, and gram staining at the microbiology laboratory of Ahmadu Bello University Teaching Hospital, Zaria, Nigeria.

### Laboratory experiments

The nasal swabs were inoculated onto mannitol salt agar (HiMedia Laboratories, Maharashtra, India). After incubation (24 h at 37 ºC), a colony was collected from each sample. The colony was reisolated to obtain a pure *S. aureus* culture for identification. A rapid in-house tube coagulation reaction and production of bright (strong) yellow colours from mannitol fermentation were used to identify the *S. aureus* strains. Ecologically, *S. aureus* is the only coagulase-positive *Staphylococcus* that readily colonises the nostrils of humans.^15^ Other coagulase-positive staphylococci are specifically adapted to dogs, cats, and horses.^16^ Furthermore, other coagulase-positive staphylococci (such as *Staphylococcus delphini, Staphylococcus ursi, Staphylococcus coagulans*, and *Staphylococcus cornubiensis*) do not ferment mannitol, while *Staphylococcus pseudintermedius* and, *Staphylococcus intermedius* weakly ferment mannitol after a prolonged incubation of > 24 h.^17^ Hence, the use of tube coagulation tests in addition to strong mannitol fermentation was considered sufficient to identify *S. aureus* in healthcare students.

The susceptibility of all identified *S. aureus* strains to different antimicrobial agents was performed using Kirby-Bauer’s disk diffusion method on Mueller-Hinton agar (Oxoid, Manchester, United Kingdom). This was done using the following Oxoid^®^ antibiotic discs (µg/disc): penicillin (1), cefoxitin (30), mupirocin (200), gentamicin (10), clindamycin (2), erythromycin (15), ciprofloxacin (5), chloramphenicol (30), tetracycline (30), and linezolid (10). The 2024 European Committee on Antimicrobial Susceptibility Testing criteria were used for breakpoint interpretation.^18^ Methicillin resistance was determined by the identification of the cefoxitin resistance phenotype on the Mueller-Hinton agar plate. For epidemiological purposes, the MAR index was defined as the number of antibiotics to which an isolate was resistant, divided by the total number of antibiotics tested. *Staphylococcus aureus* strains with MAR indices of greater than two (> 2) were used to indicate areas with high antibiotic pressure, as previously described.^19^ Furthermore, an isolate was considered MDR if it was resistant to ≥ 3 classes of the antimicrobial agents tested.^20^

### Data analysis

EpiInfo® (Centers for Disease Control and Prevention, Atlanta, United States) was used to digitise data collected on paper questionnaires. Categorical sociodemographic variables were expressed as frequencies and compared with the detection rates of nasal *S. aureus* using the Chi-square (χ^2^) test. Furthermore, bivariate analyses were performed to determine the association between risk factors and sociodemographic variables with *S. aureus* and MDR-*S. aureus* carriage rates by MedCalc Version 23.0.2 (MedCalc, Ostend, Belgium). All analysis outputs with *p* < 0.05 at 95% confidence interval were considered statistically significant.

## Results

The 251 medical and health science students were enrolled in different undergraduate programmes. The age range of the participants was from 17 years to 44 years with a mean ± standard deviation of 23.1 ± 3.5. About 50.2% were men and 124 of the students were in the clinical phase of training (exposed to healthcare settings). Most of the students in their clinical phase were in their first year (*n* = 50, 40.3%), followed by those in their third year (*n* = 39, 31.5%) (Table 1).

**TABLE 1:** Sociodemographic variables of study participants in Zaria, Nigeria, January 2024 to July 2024 (*N* = 251).

Variables	*n*	%
**Sex**		
Male	126	50.2
Female	125	49.8
**Age range (years)**		
16–21	75	29.9
22–27	167	66.5
28–33	6	1.6
34–39	2	0.8
40–45	1	0.4
**Place of residence**		
Rural	31	12.4
Suburb	35	13.9
Urban	185	73.7
**Phase of study**		
Clinical	124	49.4
Preclinical	127	50.6
**Duration of clinical posting (years)**		
0	127	50.6
1	50	19.9
2	27	10.8
3	39	15.5
4	8	3.2
**Household density (no. of people)**		
1–4	58	23.1
5–8	126	50.2
≥ 9	67	26.7
**Antibiotic usage**		
Yes	115	45.8
No	136	54.2
**Contact with pets or livestock**		
Yes	142	56.6
No	109	43.4

One hundred and eighty-five (73.7%) of the students were residing in urban settlements, 31 (12.4%) resided in rural areas, and 35 (13.9%) in suburban settlements. One hundred and twenty-six of the students resided in houses with a density of 5–8 individuals (50.2%). One hundred and fifteen (45.8%) consumed antibiotics within the last 3 months before their enrolment into the study (Table 1).

### Prevalence of *Staphylococcus aureus* and methicillin-resistant *Staphylococcus aureus* among healthcare students

**FIGURE 1:**
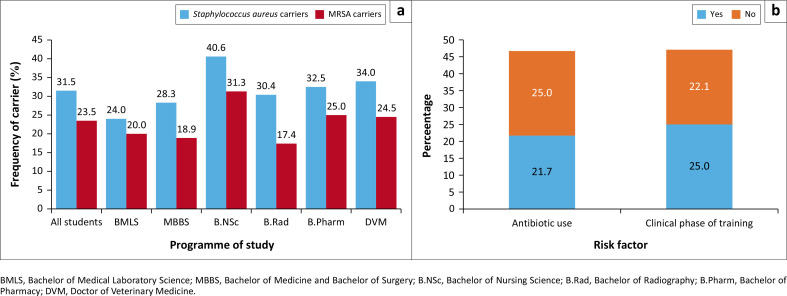
(a) Prevalence of nasal *Staphylococcus aureus* and methicillin-resistant *S. aureus* carriage and (b) association between the phase of study and antibiotic use with the prevalence of nasal methicillin-resistant *S. aureus* among among health sciences students of the Ahmadu Bello University; Zaria, Nigeria, January 2024 to July 2024.

The nasal carriage rate of *S. aureus* and MRSA among the 251 participants was 31.5% (*n* = 79) for *S. aureus* and 23.5% (*n* = 59) for MRSA (Figure 1a). Specifically, the prevalence of *S. aureus* was highest among the Bachelor of Nursing Science students (13/32, 40.6%), followed by Doctor of Veterinary Medicine (18/53, 34%), Bachelor of Pharmacy (3/40, 32.5%), Bachelor of Radiography students (7/23, 30.4%), Bachelor of Medicine, Bachelor of Surgery (MBBS) (15/53, 28.3%), and Bachelor of Medical Laboratory Science students (12/50, 24%). Furthermore, the prevalence of MRSA was highest among the Bachelor of Nursing Science (10/32, 31.3%), followed by Bachelor of Pharmacy (10/40, 25%), Doctor of Veterinary Medicine (13/53, 24.5%), Bachelor of Medical Laboratory Science (10/50, 20%), MBBS students (10/53, 18.9%), and Bachelor of Radiography students (4/23, 17.4%). Students in the clinical phase had a relatively higher frequency of nasal MRSA (31/124, 25%) than those in the preclinical phase (28/127, 22.1%) (odds ratio = 1.1786, *p* = 0.5814) (Figure 1b). There was no statistically significant association between the nasal carriage rate of *S. aureus* (*χ*^2^ = 2.97, *p* = 0.705) and MRSA (*χ*^2^ = 6.695, *p* = 0.2443) with the programme of study of the participants (*p* > 0.05) (Figure 1b).

### Prevalence and factors associated with nasal *Staphylococcus aureus* carriage

The frequency of nasal *S. aureus* carriage in female students (33/126, 32.8%) was relatively higher than in male students (41/125, 30.2%). Students within the age range of 28–33 years had the highest prevalence of nasal *S. aureus* (4/6, 66.7%), followed by those 16–21 years old (28/75, 37.3%), and those 22–27 years old (47/167, 28.1%). None of the students within the 34–45 range had *S. aureus* carriage (Table 2). Students aged 28–33 years had the highest odds of nasal *S. aureus* carriage (odds ratio = 5.106, 95% confidence interval: 0.9047–28.82) (Table 2). Students who resided in rural areas had the highest prevalence of nasal *S. aureus* (13/31, 41.9%), followed by those in the suburbs (12/35, 34.3%) and those in urban settlements (54/185, 29.2%). Students in the preclinical phase of their training had a relatively higher frequency of nasal *S. aureus* carriage (41/127, 32.3%) than those in the clinical phase (38/124, 30.6%). Of those in the clinical phase of their training, those who had been on clinical and/or laboratory posting for 3 years (15/39, 38.5%) had the highest prevalence of *S. aureus,* while the lowest was recorded among students who had been on posting for 1 year (12/50, 24%) (Table 2). Students who resided in houses with densities of 5–8 individuals (51/75, 40.7%) had the highest prevalence of *S. aureus*, followed by those who resided in houses with ≥ 9 individuals (16/51, 23.9%) (Table 2). Students who resided in houses with a density of 5–8 individuals had statistically significant odds of nasal *S. aureus* carriage (odds ratio = 0.3836, 95% confidence interval: 0.19–0.81, *p* = 0.0086) (Table 2).

**TABLE 2:** Prevalence of *Staphylococcus aureus* culture-positive and factors associated with nasal carriage in the healthcare students in Zaria, Nigeria, January 2024 to July 2024.

Variables	Culture +	Culture -	Prevalence	%	OR	CI	*p*
**Sex**							
Male	38	88	33/129	30.2	Reference	-	-
Female	41	84	41/125	32.8	0.8847	0.5243–1.485	0.6523
**Age range (years)**							
16–21	28	47	28/75	37.3	1.521	0.8544–2.708	0.1541
22–27	47	120	47/167	28.1	Reference	-	-
28–33	4	2	4/6	66.7	5.1064	0.9047–28.82	0.0648
34–39	0	2	0/2	0	-	-	-
40–45	0	1	0/1	0	-	-	-
**Place of residence**							
Rural	13	18	13/31	41.9	1.752	0.8114–3.827	0.1556
Suburb	12	23	12/35	34.3	1.266	0.5834–2.611	0.5463
Urban	54	131	54/185	29.2	Reference	-	-
**Phase of study**							
Clinical	38	86	38/124	30.6	Reference	-	-
Preclinical	41	86	41/127	32.3	0.9268	0.5494–1.556	0.7799
**Duration of clinical posting (years)**							
0	41	86	41/127	32.3	1.628	0.7589–3.671	0.2105
1	12	38	12/50	24	Reference	-	-
2	10	17	10/27	37	2.032	0.7532–5.421	0.1735
3	15	24	15/39	38.5	2.159	0.8906–5.661	0.1019
4	2	6	2/8	25	1.152	0.2120–5.923	0.8733
**Household density (no. of people)**							
1–4	12	46	12/46	20.7	Reference	-	-
5–8	51	75	51/75	40.7	0.3836	0.1880–0.8082	0.0086*
≥ 9	16	51	16/51	23.9	0.8315	0.3732–1.914	0.6696
**Antibiotic usage**							
Yes	36	79	36/115	31.3	Reference	-	-
No	43	93	43/136	31.6	0.9856	0.5805–1.656	0.9575
**Contact with pets or livestock**							
Yes	41	101	41/142	28.9	Reference	-	-
No	38	71	38/109	34.9	0.7585	0.4489–1.285	0.3112

+, positive; -, negative; OR, odds ratio; CI, confidence interval.

* , Significantly higher risk (*p* < 0.05) of nasal *Staphylococcus aureus* carriage at 95% confidence interval.

Students who used antibiotics within the last 3 months before their enrolment into the study had a closely similar frequency of nasal *S. aureus* carriage (31.6%) compared with those with no antibiotic use 3 months before enrolment in the study (31.3%). Students who had prolonged contact with pets or livestock had a relatively lower frequency of nasal *S. aureus* carriage (41/142, 28.9%), than those who did not (38/109, 34.9%) (Table 2).

### Factors associated with nasal carriage of multidrug resistant-*Staphylococcus aureus*

The frequency of multidrug resistant-*S. aureus* was highest among Bachelor of Pharmacy students (9/14, 64.3%), followed by MBBS (7/15, 46.7%), Doctor of Veterinary Medicine (7/18, 38.9%), and Bachelor of Nursing Science students (5/13, 38.5%). The frequency of nasal MDR-*S. aureus* in female students (19/41, 46.3%) was relatively higher than in male students (12/38, 31.6%). Students within the age range of 22–27 years had the highest prevalence of nasal MDR-*S. aureus* (19/47, 40.4%), followed by those 16–21 years (11/28, 39.2%), and those 28–33 years, (1/4, 25%) (Table 3). Students who resided in suburban areas had the highest prevalence of nasal MDR-*S. aureus* (6/12, 50%), followed by those living in rural areas (5/13, 38.5%), and those in urban settlements (20/54, 37%). Students in the clinical phase of their training had a closely similar frequency of nasal MDR-*S. aureus* carriage (15/38, 39.4%) to those in the preclinical phase (16/41, 39%). Of those in the clinical phase of their training, those that had been on clinical/laboratory posting for 4 years (1/2, 50%) had the highest frequency of MDR-*S. aureus*, while the lowest was recorded in students who had been on posting for 2 years (3/10, 30%) (Table 3). Students who resided in houses with densities of 5–8 individuals (24/51, 47.1%) had the highest frequency of MDR-*S. aureus*, followed by those that resided in houses with 1–4 individuals (4/12, 33.9%). Students who used antibiotics within the last 3 months before their enrolment into the study had a relatively higher frequency of nasal MDR-*S. aureus* carriage (44.4%), compared with those with no antibiotic use 3 months before enrolment in the study (34.9%). Students who had prolonged contact with pets or livestock had a relatively higher frequency of nasal MDR-*S. aureus* carriage (17/41, 41.5%) than those who did not (14/38, 36.8%). Both MBBS and Bachelor of Pharmacy students had statistically significant high frequencies of nasal MDR-*S. aureus* (both *p* < 0.05). Furthermore, students who resided in houses with a density of 5–8 individuals had the highest odds of nasal MDR-*S. aureus* carriage (odds ratio = 3.85, 95% confidence interval: 0.99–13.66, *p* = 0.0044) (Table 3).

**TABLE 3:** Frequency of culture-positive MDR-*Staphylococcus aureus* and factors associated with its nasal carriage in the healthcare students in Zaria, Nigeria, from January 2024 to July 2024.

Variables	Culture +	Culture -	Prevalence		OR	CI	*p*
			*n*/*N*	%			
**Programme of study**							
BMLS	1	11	1/12	8.3	Reference	-	-
MBBS	7	8	7/15	46.7	0.1039	0.008583–0.9554	0.0302*
BNSc	5	8	5/13	38.5	0.1455	0.01148–1.109	0.078
BRad	2	5	2/7	28.6	0.2273	0.01460–2.502	0.2432
BPharm	9	5	9/14	64.3	0.05051	0.004302–0.4818	0.0035*
DVM	7	11	7/18	38.9	0.1429	0.01178–1.220	0.0637
**Sex**							
Male	12	26	12/38	31.6	Reference	-	-
Female	19	22	19/41	46.3	0.5344	0.2110–1.399	0.1794
**Age range (years)**							
16–21	11	17	11/28	39.2	1.941	0.2551–27.24	0.5809
22–27	19	28	19/47	40.4	2.0357	0.1967–21.07	0.5511
28–33	1	3	1/4	25	Reference	-	-
34–39	0	1	0/1	0	-	-	-
40–45	0	1	0/1	0	-	-	-
**Place of residence**							
Rural	5	8	5/13	38.5	1.063	0.3326 – 3.432	0.924
Suburb	6	6	6/12	50	1.7	0.4865 – 5.707	0.4058
Urban	20	34	20/54	37	Reference	-	-
**Phase of study**							
Clinical	15	23	15/38	39.4	Reference	-	-
Preclinical	16	25	16/41	39	1.019	0.4314 – 2.394	0.9674
**Duration of clinical posting (years)**							
0	16	25	16/41	39	1.493	0.3477–5.878	0.5966
1	5	6	5/11	45.5	1.944	0.3735–9.567	0.4664
2	3	7	3/10	30	Reference	-	-
3	6	9	6/15	40	1.556	0.2571–7.161	0.6098
4	1	1	1/2	50	2.333	0.09374–49.78	0.5839
**Household density (no. of people)**							
1–4	4	8	4/12	33.3	2.167	0.4507–10.13	0.3778
5–8	24	27	24/51	47.1	3.852	0.9882–13.66	0.044*
≥ 9	3	13	3/16	18.8	Reference	-	-
**Antibiotic usage**							
Yes	16	20	16/46	44.4	1.493	0.6279–3.588	0.3861
No	15	28	15/43	34.9	Reference	-	-
**Contact with pets or livestock**							
Yes	17	24	17/41	41.5	1.214	0.5088–2.911	0.6743
No	14	24	14/38	36.8	Reference	-	-

+, positive; -, negative; OR, odds ratio; CI, confidence interval; BMLS, Bachelor of Medical Laboratory Science; MBBS, Bachelor of Medicine and Bachelor of Surgery; BNSc, Bachelor of Nursing Science; BRad, Bachelor of Radiography; BPharm, Bachelor of Pharmacy; DVM, Doctor of Veterinary Medicine.

* , Significantly higher risk (*p* < 0.05) of nasal MDR-*Staphylococcus aureus* carriage at 95% confidence interval.

### Antimicrobial resistance rates in *Staphylococcus aureus* isolates

**FIGURE 2:**
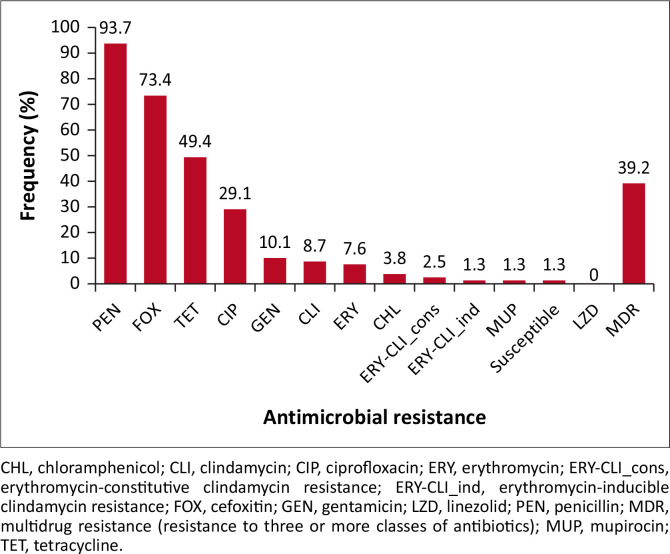
Antimicrobial resistance rates in 79 *Staphylococcus aureus* strains obtained from the participants in Zaria, Nigeria, January 2024 to July 2024.

Out of the 79 *S. aureus* strains identified, the pattern of resistance to tested antimicrobial agents, in descending frequency, was as follows: penicillin (93.7%), cefoxitin (73.4%), tetracycline (49.4%), ciprofloxacin (29.1%), gentamicin (10.1%), clindamycin (8.7%), erythromycin (7.6%), chloramphenicol (3.8%), erythromycin-constitutive clindamycin (2.5%), erythromycin-inducible clindamycin resistance (1.3%), high-level mupirocin (1.3%). All strains were susceptible to linezolid, and one strain (1.3%) was completely susceptible to all the antibiotics tested (Figure 2). About 39.2% of strains presented the MDR phenotype, of which one strain presented an erythromycin-inducible clindamycin resistance (Figure 2). Over 30% of the *S. aureus* strains had similar MDR phenotypes (Table 2).

**FIGURE 3:**
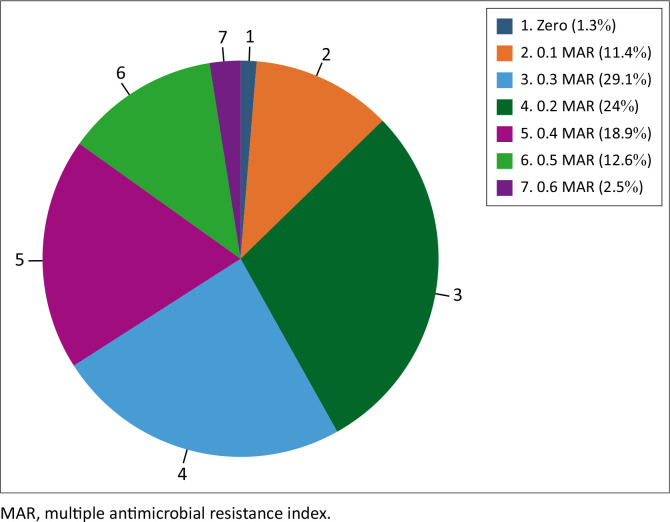
Levels and frequency of multiple antimicrobial resistance indices of *Staphylococcus aureus* isolates in Zaria, Nigeria, January 2024 to July 2024.

Eight (10.1%) of the *S. aureus* strains were solely resistant to penicillin, and one (1.3%) to cefoxitin, with a MAR index of 0.1 (Table 4). Two strains had the highest antimicrobial resistance profile, with a MAR index of 0.6 for resistance to penicillin-cefoxitin-tetracycline-clindamycin-gentamicin-ciprofloxacin and penicillin-cefoxitin-tetracycline-erythromycin-clindamycin-ciprofloxacin (Table 4). Strains with a MAR index of 0.2 were the most predominant (29.1%), followed by strains with a MAR index of 0.3 (24%), 0.4 (18.9%), 0.5 (12.6%), and 0.6 (2.5%) (Figure 3).

**TABLE 4:** Antimicrobial resistance profile and multiple antimicrobial resistance indices of the *Staphylococcus aureus* isolates from the study participants in Zaria, Nigeria, January 2024 to July 2024.

Number of strains	Antimicrobial resistance phenotypes	MRSA	MAR index	MDR
1	Susceptible	No	0	No
8	PEN	No	0.1	No
1	FOX	Yes	0.1	No
17	PEN-FOX	Yes	0.2	No
5	PEN-TET	No	0.2	No
1	PEN-GEN	No	0.2	No
8	PEN-FOX-TET	Yes	0.3	No
1	PEN-FOX-CLI	Yes	0.3	No
5	PEN-FOX-CIP	Yes	0.3	No
1	PEN-FOX-ERY	Yes	0.3	No
2	PEN-TET-CIP	No	0.3	Yes
2	PEN-TET-CLI	No	0.3	Yes
1	PEN-FOX-TET-GEN	Yes	0.4	Yes
3	PEN-FOX-TET-CLI	Yes	0.4	Yes
8	PEN-FOX-TET-CIP	Yes	0.4	Yes
3	PEN-FOX-TET-ERY	Yes	0.4	Yes
1	PEN-FOX-TET-CLI-MUP	Yes	0.5	Yes
2	PEN-FOX-TET-GEN-CIP	Yes	0.5	Yes
2	PEN-FOX-TET-ERY-GEN	Yes	0.5	Yes
1	PEN-FOX-ERY-CLI-CIP	Yes	0.5	Yes
2	PEN-FOX-ERY-CLI^cons^-CHL	Yes	0.5	Yes
1	PEN-FOX-TET-CHL-CIP	Yes	0.5	Yes
1	PEN-FOX-TET-ERY-CIP	Yes	0.5	Yes
1	PEN-FOX-TET-ERY-CLI^ind^-GEN-CIP	Yes	0.6	Yes
1	PEN-FOX-TET-ERY-CLI^cons^-CIP	Yes	0.6	Yes

CHL, chloramphenicol; CLI, clindamycin; CIP, ciprofloxacin; ERY, erythromycin; ERY-CLI^cons^, erythromycin-constitutive clindamycin; ERY-CLI^ind^, erythromycin-inducible clindamycin resistance; FOX, cefoxitin; GEN, gentamicin; LZD, linezolid; PEN, penicillin; MDR, multidrug resistance (resistance to three or more classes of antibiotics); MUP, mupirocin; TET, tetracycline; MRSA, methicillin-resistant *Staphylococcus aureus*; MAR, multiple antimicrobial resistance.

## Discussion

An overall prevalence of 31.5% for carriage of *S. aureus* and 23.5% for carriage of MRSA were obtained in this study. Subgroup analysis showed that nursing science students had the highest prevalence of both *S. aureus* and MRSA carriage. To our knowledge, this is the first epidemiological study on the MAR indices of nasal *S. aureus* colonisation rate in Nigeria. The 31.5% prevalence of *S. aureus* recorded in this study is similar to the results of studies done among healthcare students identified in medical and health science students in Plateau and Delta states of Nigeria in 2018 (37.3%) and 2024 (38.5%).^21,22^ However, the finding from the present study is similar to the nasal *S. aureus* carriage reported in other countries such as Nepal in 2011 (35%), and Poland in 2018 (30%).^23,24^ A relatively lower prevalence of 16.8% was reported in medical students at Kaduna State University (Nigeria) in 2024, and 14% at Babcock University (Nigeria) in 2020.^25,26^ Furthermore, lower prevalence values were reported in China (23.1%), Thailand (29.7%), Ethiopia (27.1%), Turkey (21.4%), Germany (22.3%) and Saudi Arabia (25.3%) from 2011 to 2022.^27,28,29,30,31,32^ Much higher nasal *S. aureus* carriage was reported by Ogefere and Ogunleye (54.7%)^33^ and Solomon et al. (66.6%),^34^ both in 2019 in Nigeria, and 50% by Rosales-González et al.^12^ in 2024 in Spain. The variations observed in the nasal *S. aureus* carriage from these studies could be because of differences in the study protocols, sample size, laboratory methods for *S. aureus* identification, and levels of antibiotic use by the students.

In the present study, there were higher odds of nasal *S. aureus* carriage with age and household density of the students. A similar finding was reported in China in 2017, where medical students of the age group 20–30 years were found to have a higher nasal *S. aureus* carriage.^35^ It was previously reported that the nasal *S. aureus* carriage tends to increase with age and population density.^36^ Households and communities in congested conditions are more likely to be exposed to *S. aureus* colonisation, with an increased transmission rate.^37,38^

Although not statistically significant, there was a trend for higher *S. aureus* colonisation in the nasal cavity in female students, and those who resided in rural settlements. A similar study in Madagascar previously reported higher *S. aureus* nasal carriage in female medical students and healthcare workers in 2016.^39^ The higher rate in female students could be because of biological reasons or differences in susceptibility to *S. aureus* colonisation as a result of circulating steroid hormones.^40^ The higher *S. aureus* rate among students who were rural residents could be because of the relatively higher contact rate of the students with animals in the rural areas, and poor personal and environmental hygiene that could facilitate the increased transmission of *S. aureus.*^41^ This is similar to the findings of Ansari et al.^42^ in 2016, who reported a higher nasal *S. aureus* carriage rate in medical students who were rural residents than in their urban counterparts.

The overall prevalence of MRSA recorded in this study is 22.7%, and comparable to 26.2% (Delta state of Nigeria), and 27.1% (Ethiopia).^22,31^ However, a higher prevalence of nasal MRSA (48.8%) was reported by Solomon et al.,^34^ while a lower value (12.2%) was reported by Rosales-González et al.^12^ Several factors have been linked to increased *S. aureus* carrier status, including sinusitis, antimicrobial treatments, prolonged contact with animals, smoking, and contact with healthcare professionals.^29,43,44^

Although not statistically significant, nasal MRSA carriage was higher in students with antibiotic usage in the previous 3 months. This was similar to studies in Ethiopia (2023) and Malaysia (2012) that found that recent antibiotic usage was associated with higher nasal carriage of MRSA.^45,46^ Furthermore, students who reported prolonged contact with animals (pets or livestock) had a relatively higher frequency of nasal MDR-*S. aureus* carriage than those who did not. This finding is not unexpected, as occupational or companionship contact with animals is a major risk factor for MDR-*S. aureus.*^47^

It is important to remark that the prevalence of MDR-*S. aureus* was statistically significantly higher in pharmacy and MBBS students. Similar findings were reported by Obajuluwa, Parom and Kubau (2024) in Nigeria.^25^ Moreover, MDR-*S. aureus* was significantly associated with households with 5–8 individuals. These reflect the degree of contact with patients in the clinical phase of training, as students who had 4 years in clinical posting had the highest MDR-*S. aureus* carriage. This finding indicates that once healthcare students start interacting directly with patients in a clinical setting, their likelihood of carrying MDR-*S. aureus* is much higher compared to before their clinical exposure as a result of the increased contact with other potential MDR-*S. aureus* carriers in healthcare settings, such as healthcare professionals (especially physicians and nurses), patients, and contaminated environment surfaces and equipment. Furthermore, high household density can facilitate the transmission of MDR strain because of increased contact with many individual carriers within the same rooms and houses. The strong antibiotic selective pressure among congested populations produces a suitable condition that could facilitate the emergence and efficient dissemination of many MDR bacteria, such as MDR-*S. aureus.*^48^

Generally, up to 30% of the human population is asymptomatic and permanently colonised with *S. aureus* in their nostrils.^49^ However, MDR-*S. aureus* strains, especially MRSA, can be translocated from the nose to other parts of the body, causing infection or facilitating transmission to other humans, animals, and the environment through nasal secretion droplets during sneezing, coughing, kissing, or handshaking.^50,51,52^

Most of the *S. aureus* isolates were resistant to penicillin and ciprofloxacin. This could be explained by the widespread and indiscriminate use of these classes of antibiotics in the treatment of *S. aureus.*^49^ Similar findings were found in many other studies in Nigeria (2011 to 2024).^24,25,34,53^ The presence of mupirocin-resistant *S. aureus* (1.4%) may render mupirocin less effective as a nasal decoloniser or as topical treatment for superficial skin infections.^54^

Fortunately, linezolid resistance was not detected in any of the *S. aureus* strains and very low resistance (only one student) to chloramphenicol was recorded. However, tetracycline resistance was high. This suggests that our research participants had prior contact with livestock, as tetracycline resistance is more frequent in livestock breeders than in healthy humans with no occupational contact with livestock.^55^ This is because tetracycline is a very common antimicrobial agent used in livestock husbandry, prophylaxis, and as a first-line treatment of most bacterial infections. Consequently, bacteria (including *S. aureus*) found in farm settings (including farmers) exhibit high selective pressure against tetracycline.^55^

Remarkably, one strain presented a characteristic erythromycin-inducible clindamycin resistance. This phenotypic feature could be useful as a marker for the surveillance of *S. aureus* CC398 lineage.^55^ However, this finding is subject to further molecular confirmation, such as the screening for the *ermT* and *ermC* genes.^55,56^

Multidrug resistance was high in the *S. aureus* carriers, as some isolates presented resistance against five or six classes of antibiotics (MAR index ≥ 5). Therefore, MDR-*S. aureus* may limit the available chemotherapeutic options against staphylococcal and many other Gram-positive bacterial infections.^53^ This shows that most of the *S. aureus* strains originated from areas with high antibiotic pressure.^16^

### Recommendations

First, it is recommended to consider the improvement of infection prevention and control measures in the wards, clinics, laboratories, pharmacies, and veterinary hospitals where healthcare students undertake training. Furthermore, the implementation of active hospital and antimicrobial resistance surveillance systems should include all medical, and other groups of healthcare students as potential reservoirs of nasal MDR-*S. aureus* and MRSA.

Second, it will be interesting to determine the clonality of MRSA strains with similar antimicrobial resistance profiles from different students. Hence, pulse-field gel electrophoresis and/or whole phylogenomic analysis could provide insight into the potential transmission route of these strains.

### Limitations

This study is not without limitations. First, the use of phenotypic identification of antimicrobial resistance using disk diffusion may not account for some antimicrobial agents that could be phenotypically susceptible when tested by the disk diffusion method but could still carry the antimicrobial resistance genes. For instance, linezolid resistance is best identified by the determination of minimum inhibition concentrations through broth dilution tests or *E*-tests. Second, the use of automated bacterial identification systems such as Bactec and Vitek II compact, and advanced equipment such as the Matrix-assisted laser desorption ionisation-time of flight (mass spectrometry could have allowed the possibility to determine the performance characteristics of combined tube coagulase test and strong mannitol fermentation in the identification of *S. aureus.*

### Conclusion

High levels of nasal MRSA and MDR-*S. aureus* were obtained from this study. The predominance of strains with high MAR indices indicates that strains were from locations with much antibiotic pressure. Furthermore, many strains with similar antimicrobial resistance profiles could indicate potential interhost transmission within human clusters either at the community level or clinics and laboratories where these students were performing their postings. Collectively, these students could serve as vectors of transmission of MDR and virulent strains to the patients or the community. Based on this, it could be inferred that the medical and health science students are a potential source of MRSA strains at Ahmadu Bello University, Zaria, Nigeria.^7^
